# Evaluation of the Antiseizure Activity of Endemic Plant *Halfordia kendack* Guillaumin and Its Main Constituent, Halfordin, on a Zebrafish Pentylenetetrazole (PTZ)-Induced Seizure Model

**DOI:** 10.3390/ijms24032598

**Published:** 2023-01-30

**Authors:** Adrianna Skiba, Ewelina Kozioł, Simon Vlad Luca, Barbara Budzyńska, Piotr Podlasz, Wietske Van Der Ent, Elham Shojaeinia, Camila V. Esguerra, Mohammed Nour, Laurence Marcourt, Jean-Luc Wolfender, Krystyna Skalicka-Woźniak

**Affiliations:** 1 Department of Natural Products Chemistry, Medical University of Lublin, 20-093 Lublin, Poland; 2 Biothermodynamics, TUM School of Life Sciences, Technical University of Munich, 85354 Freising, Germany; 3 Department of Pharmacognosy, Grigore T. Popa University of Medicine and Pharmacy Iasi, 700115 Iasi, Romania; 4 Independent Laboratory of Behavioral Studies, Medical University, Chodzki 4a, 20-090 Lublin, Poland; 5 Department of Pathophysiology, Forensic Veterinary and Administration, Faculty of Veterinary Medicine, University of Warmia and Mazury, 10-719 Olsztyn, Poland; 6 Chemical Neuroscience Group, Centre for Molecular Medicine Norway (NCMM), University of Oslo, Forskningsparken, Gaustadalleén 21, 0349 Oslo, Norway; 7 Department of Pharmacy, Section for Pharmacology and Pharmaceutical Biosciences, Faculty of Mathematics and Natural Sciences, University of Oslo, Blindern, P.O. Box 1068, 0316 Oslo, Norway; 8 Institut des Sciences Exactes et Appliquées (ISEA)-EA 4243, France University of New Caledonia, 98851 Nouméa, New Caledonia, France; 9 School of Pharmaceutical Sciences, University of Geneva, CMU, Rue Michel Servet 1, 1211 Geneva, Switzerland; 10 Institute of Pharmaceutical Sciences of Western Switzerland, University of Geneva, CMU, Rue Michel Servet 1, 1211 Geneva, Switzerland

**Keywords:** liquid-liquid chromatography, coumarins, zebrafish, epilepsy, LFP, electrophysiology, rutaceae

## Abstract

Epilepsy is a neurological disease that burdens over 50 million people worldwide. Despite the considerable number of available antiseizure medications, it is estimated that around 30% of patients still do not respond to available treatment. Herbal medicines represent a promising source of new antiseizure drugs. This study aimed to identify new drug lead candidates with antiseizure activity from endemic plants of New Caledonia. The crude methanolic leaf extract of *Halfordia kendack* Guillaumin (Rutaceae) significantly decreased (75 μg/mL and 100 μg/mL) seizure-like behaviour compared to sodium valproate in a zebrafish pentylenetetrazole (PTZ)-induced acute seizure model. The main coumarin compound, halfordin, was subsequently isolated by liquid-liquid chromatography and subjected to locomotor, local field potential (LFP), and gene expression assays. Halfordin (20 μM) significantly decreased convulsive-like behaviour in the locomotor and LFP analysis (by 41.4% and 60%, respectively) and significantly modulated *galn*, and *penka* gene expression.

## 1. Introduction

Epilepsy is one of the most common neurological diseases affecting approximately 50 million people worldwide. It is characterised by seizures that might be limited to one brain hemisphere (partial) or the entire brain (generalised). The most well-known symptom of epilepsy is a visible seizure, which may be accompanied by a loss of consciousness.

Sometimes, seizures are accompanied by a loss of control over the bowels and bladder, which may lead to social stigma, increased stress, anxiety, and depression. Even though over 25 antiseizure medications (ASMs) are approved worldwide, around 25–30% of patients do not respond to treatment or experience numerous side effects, such as gastrointestinal problems, hepatotoxicity, or cognitive impairments. Therefore, discovering new, efficient, and safe drugs is highly important [1,2].

Medicinal plants are a rich source of valuable compounds with diverse bioactivity, ranging from cardioprotective to anti-proliferative, as well as affecting central nervous system (CNS) [3,4,5]. Since they have been used for decades in traditional medicine, they are usually acknowledged for their safety and efficacy. Yet, many promising plants have not been screened for their chemical composition or bioactivity. This is the case for plants endemic to Australia, New Guinea, or New Caledonia, especially from the Rutaceae family. In a previous investigation from our group [6], rare alkaloids from *Medicosma leratii* (Guillaumin) T.G. Hartley collected in New Caledonia were isolated and shown to possess interesting pro-apoptotic properties in in vitro assays [6].

*Halfordia kendack* Guillaumin (saffron heart) is another species of Rutaceae family distributed in the rain forests of Australia, New Guinea, and New Caledonia. Previous phytochemical investigations on *H. kendack* revealed that the plant contains rare coumarins (e.g., furanocoumarins—halfordin) and quinolone alkaloids (e.g., *trans*-erioaustrasine, *trans*-deacetoxyarioaustrasine, *trans*-australasine hydrate) [7,8]. Alkaloids and coumarins with similar structures to those found in *H. kendack* (e.g., imperatorin, xanthotoxin, skimmianine) were already reported in the literature as antidepressant, neuroprotective, antiseizure, and pro-cognitive agents [9,10,11,12,13,14,15].

Thus, as a continuation of our research aiming to find new, safe, and effective drug-like molecules derived from natural sources, the locomotor activity of a crude methanol leaf extract of *H. kendack* was evaluated in a zebrafish pentylenetetrazole (PTZ)-induced seizure model. It is a model of acute, generalised, tonic-clonic type of seizures due to inhibition of ɣ-aminobutyric acid (GABA) type A (GABA_A_) receptor and a well-established model for screening small molecules with potent antiseizure activity [16]. It is similar to the rodent seizure model; however, due to the high fertility of fish and the small size of larvae, it might be used in high-throughput assays. Seizures induced by PTZ might be evaluated by behavioural, electroencephalogram (EEG), and gene expression changes [17,18,19]. Since the crude extract exhibited a promising decrease in locomotor activity, the main furanocoumarin (halfordin) was next efficiently isolated by liquid-liquid chromatography (LLC). Compared to conventional liquid-solid chromatography, LLC offers a wide range of advantages, such as high loading capacity, good selectivity, low solvent consumption, and the absence of sample loss due to the irreversible adsorption of degradation within the column [20,21]. LLC has been shown to be suitable for isolating numerous natural products from complex plant matrices [22,23]. The isolation was followed by the evaluation of the antiseizure potential of the isolated halfordin in locomotor, local field potential (LFP), and gene expression assays in the zebrafish model. Thus, combining two state-of-the-art platforms, one phytochemical (LLC) and one pharmacological (zebrafish), halfordin was isolated in high-purity from *H. kendack* and identified as a potent molecule against seizures induced by PTZ in zebrafish.

## 2. Results

### 2.1. LLC Isolation of Halfordin

**Figure 1:**
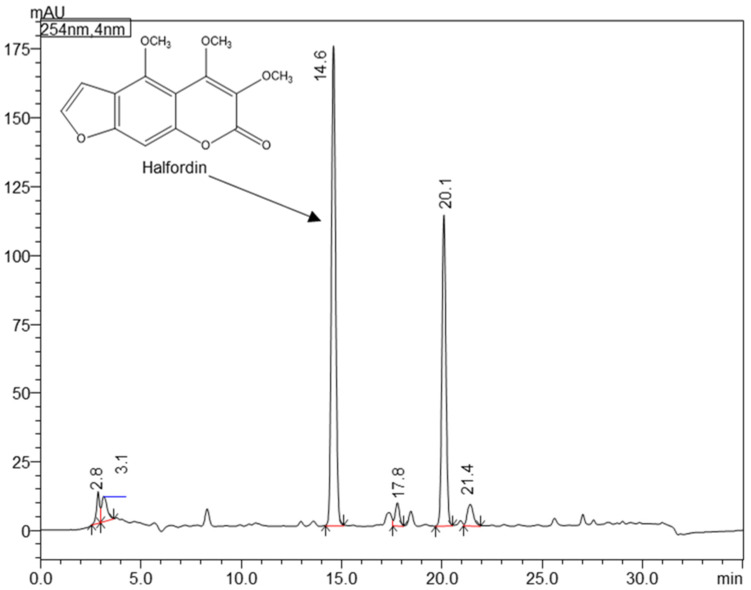
HPLC-DAD chromatogram of the *H. kendack* methanol leaf extract with the main compound halfordin marked (detection 254 nm; Phenomenex Gemini NX-C18 110A (250 mm × 4.6 mm, 5 μm, Warsaw, Poland); 1 mL/min, gradient of water and methanol. Peak rf = 14.6: halfordin, peak rf = 20.1: unidentified alkaloid.

The H. kendack methanol leaf extract was initially analysed by HPLC-DAD (Figure 1) and HPLC-DAD-ESI-QTOF-MS/MS, revealing the fingerprints of halfordin as one of the main compounds, as was already reported [7]. The suitable biphasic solvent system for the LLC isolation of halfordin was selected based on the partition coefficient (K_D_) values determined in the shake-flask experiments and presented in Table 1. According to the literature, it is recommended that the K_D_ values should range between 0.2–5 [24]. Most of the tested HEMWat volume ratios provided K_D_ values of halfordin within the pre-defined range. Therefore, HEMWat 3/1/3/1 (*v*/*v*/*v*/*v*) was selected for the LLC of the coumarins, considering that the smaller the K_D_ values, the shorter the separation time.

The LLC separation of halfordin was performed in descending mode, with the lower phase as the mobile phase. After sample injection (270 mg of crude methanol leaf extract of *H. kendack* dissolved in 10 mL of lower phase), continuous fraction collection for 50 min, and offline HPLC-DAD analysis, fractions containing halfordin (fractions 15–17) were pooled together, yielding 11.2 mg halfordin with an overall purity of >93%. The LLC chromatogram and all operating conditions are provided in Figure S1. The structure identity of halfordin was confirmed by HPLC-DAD-ESI-QTOF-MS/MS and NMR experiments (Section 4.3.6). Thus, LLC allowed the efficient and fast purification of high-purity halfordin in sufficient amounts for further bioassays.

### 2.2. Zebrafish Locomotor Assay

The *H. kendack* methanol leaf extract (25, 50, 75, and 100 μg/mL) and halfordin (5, 10, 15, and 20 μM) were tested on the acute PTZ seizure model in zebrafish, using 5 mM sodium valproate (VPN) as the positive control. 20 mM PTZ significantly increased the actinteg values, anarbitrary unit used to denote total locomotor activity of zebrafish larvae in all treated groups, confirming its proconvulsant effect (*p* < 0.001).

**Figure 2:**
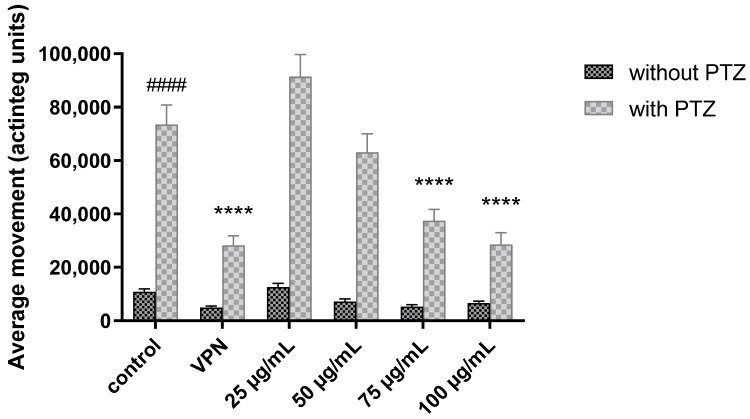
Anticonvulsant activity of *Halfordia kendack* methanol leaf extract and positive control (5 mM sodium valproate, VPN) after 18 h incubation prior to 20 mM pentylenetetrazole (PTZ) treatment (n = 30). Data represent means  ±  SEM; #### *p* < 0.0001 vs. vehicle-PTZ control group **** *p <* 0.0001 vs. PTZ-treated group (post-hoc Bonferroni test).

After 18 h of incubation, the *H. kendack* methanol leaf extract (75–100 μg/mL) reduced the convulsion effects induced by PTZ. Two-way ANOVA analysis showed a statistically significant effect in PTZ treatment [F(1,335) = 306.8; *p* < 0.0001], 5 mM VPN, and different concentrations of extract pretreatment [F(5,335) = 21.04; *p* < 0.0001], as well as interactions between treatment and pretreatment [F(5,335) = 13.58; *p* < 0.0001]. VPN and extract at the concentration 75 μg/mL (*p* < 0.0001) and 100 µg/mL (*p* < 0.0001) decreased the activity (61.6%, 49.0%, and 61.2%, respectively), in comparison with a vehicle-PTZ tested group (Figure 2).

**Figure 3:**
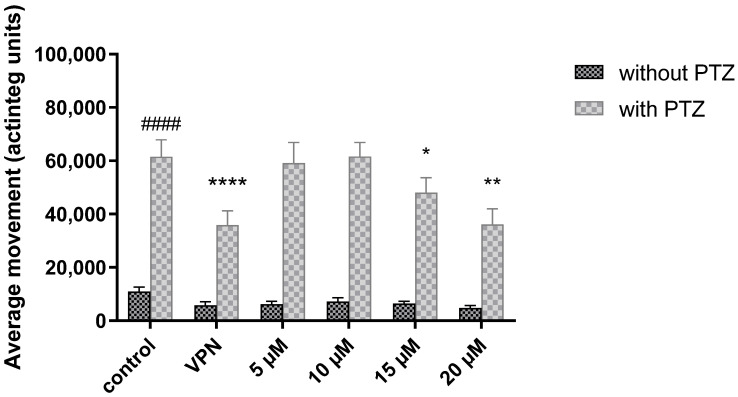
Anticonvulsant activity of halfordin (HAL) and positive control (5 mM sodium valproate, VPN) after 18 h incubation prior to 20 mM pentylenetetrazole (PTZ) treatment (n = 30). Data represent means  ±  SEM and #### *p* < 0.0001 vs. vehicle-PTZ control group **** *p <* 0.0001, ** *p* < 0.01 * *p* < 0.05 vs. PTZ-treated group (post-hoc Bonferroni test).

After 18 h of incubation, halfordin (15–20 μM) statistically reduced the convulsions induced by PTZ. Two-way ANOVA analysis showed a statistically significant effect in PTZ treatment [F(1,335) = 308.8; *p* < 0.0001], 5 mM VPN, and different concentrations of halfordin pretreatment [F(5,335) = 5.183; *p* = 0.0001], as well as interactions between treatment and pretreatment [F(5,335) = 3.204; *p* = 0.0077]. Both 5 mM VPN (*p* < 0.001) and halfordin at the concentration of 15 µM (*p* < 0.05) and 20 µM (*p* < 0.01) decreased the activity of PTZ-treated larvae (41.7%, 21.9%, and 41.4%, respectively), in comparison with a vehicle-PTZ treated group which was considered as a 100% (Figure 3).

### 2.3. LFP Recordings

**Figure 4:**
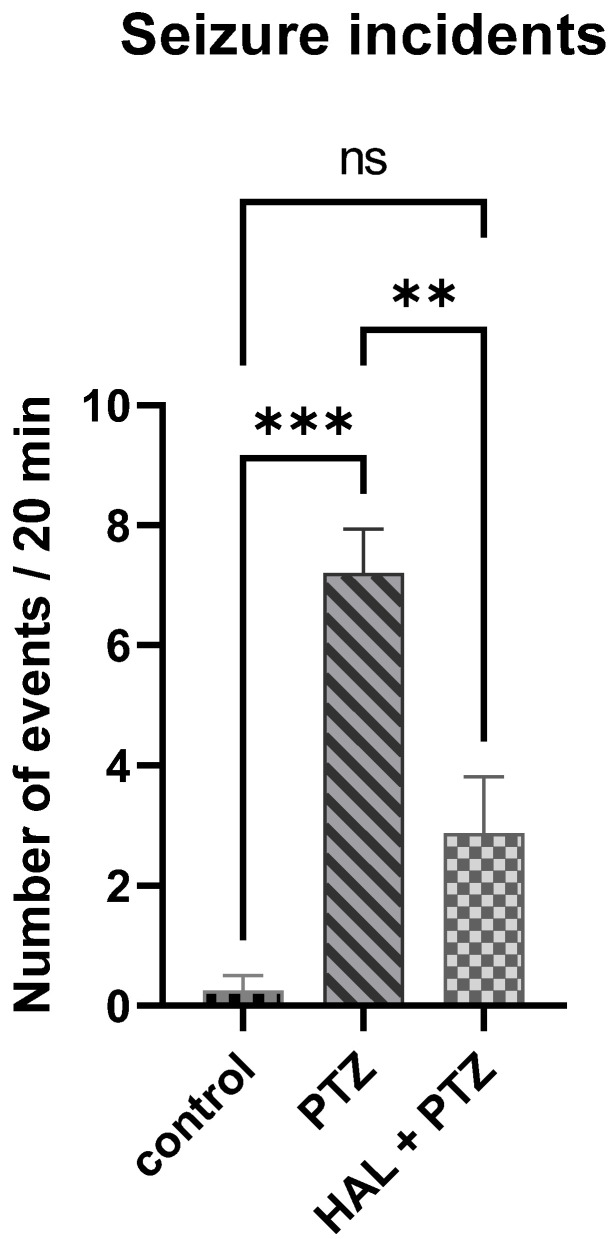
7-dpf zebrafish larvae pretreated for 18 h with 20 μM halfordin (HAL) and then 20 mM pentylenetetrazole PTZ displayed a decreased number of seizures over a 20 min period compared to those treated only with PTZ. The number of seizures observed in vehicle control is likely due to the needle insertion into the optic tectum. The one way-ANOVA with SEM and post-hoc Tukey test (*** *p* < 0.001, ** *p* < 0.01) was used to calculate the significance. ns: not significant.

To confirm the results of the locomotor activity, local field potential (LFP) recordings were performed in 8 dpf larvae. If the amplitude exceeded three times the background, the event was considered a seizure. One-way ANOVA showed a significant changes (F(2,14)  =  13.18; *p* = 0.0006). Post-hoc Tukey test confirmed that PTZ increased 29 times the number of seizure incidents (*p* < 0.001) compared to the control group. In contrast, 18 h pretreatment with halfordin at the concentration of 20 μM decreased 2.5 times the observed parameter compared with PTZ-treated larvae (*p* < 0.01). The test proved that halfordin at 20 μM reduced the seizure incidents (Figure 4). The number of seizures observed in vehicle control is likely due to the needle insertion into the optic tectum.

### 2.4. Gene Expression

**Figure 5:**
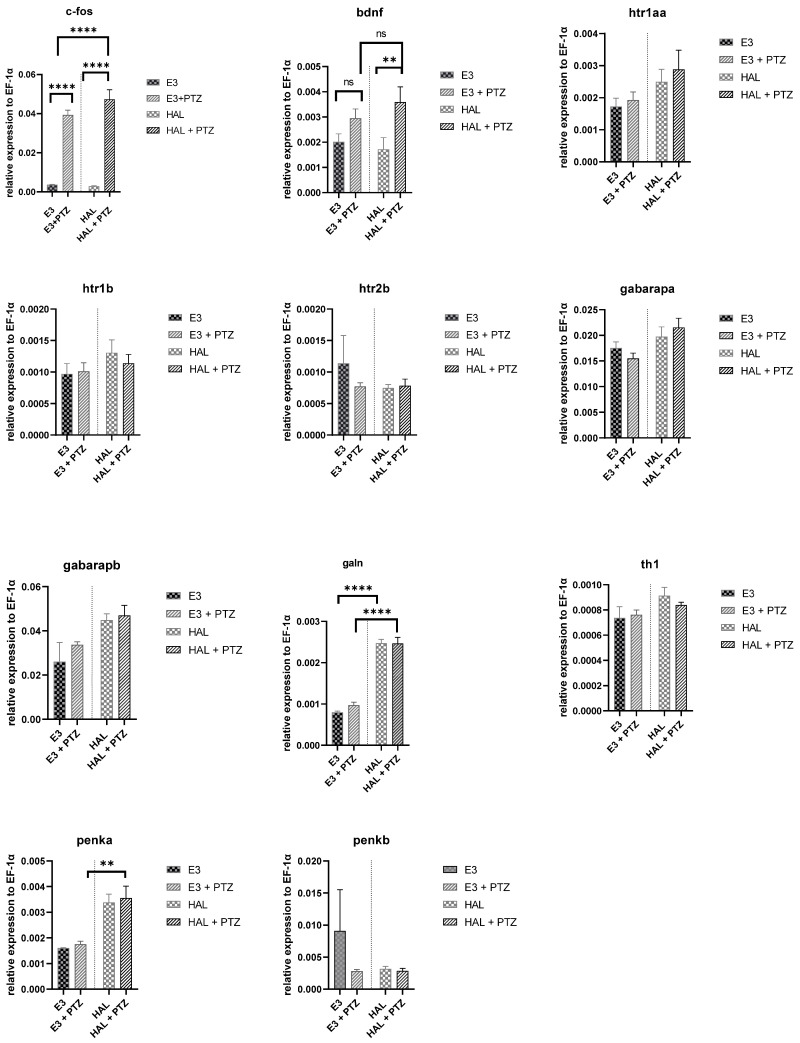
Changes of gene expression for control and halfordin-treated group before and after PTZ treatment. The one-way ANOVA with SEM and post-hoc Tukey test (** *p* < 0.01, **** *p <* 0.0001, ns: not significant) was used to calculate significance; n = 3.

To study the effect of halfordin on the expression of selected genes in the zebrafish PTZ-induced seizure model, qRT-PCR studies were performed. The expression of genes involved in epilepsy mechanisms related to the activation of neurons and neurotransmission was studied, including *c-fos*, *bdnf*, *htr1aa*, *htr1b*, *htr2b*, *gabarapa*, *gabarapb*, *galn*, *th1*, *penka*, *penkb.* There were no significant differences in the expression of most of the studied genes; only the expression of *c-fos* (a marker of neuronal activity), *bdnf*, *galn*, and *penka* was significantly altered. The expression of *c-fos* dramatically increased (over 10 fold, *p*-value < 0.0001 in the vehicle control group and 17 fold, *p*-value < 0.0001 in the group treated with halfordin) by the PTZ treatment. Halfordin treatment did not significantly change the mRNA level of *c-fos* in the control or PTZ-treated group (Figure 5).

After PTZ treatment, the expression of *bdnf* increased over two-fold (*p*-value < 0.01 for the group treated with halfordin). Halfordin treatment did not significantly change the mRNA level of *bdnf* in either the vehicle control or PTZ-treated group (Figure 5).

There were no differences after PTZ treatment in the expression of *galn*; however, significant changes were observed between the control and halfordin-treated group (an increase over three-fold, *p*-value < 0.0001). A similar situation was observed in the mRNA level of *penka* after halfordin treatment (an increase over two-fold, *p*-value < 0.01) (Figure 5).

## 3. Discussion

Halfordin was isolated for the first time from *H. kendack* by Hegarty and Lahey [25] in 1956. The isolation was preceded by 12 h Soxhlet extraction, crystallization, and column chromatography. However, the efficiency was low (3 mg of coumarin from 3 kg of plant material) [25]. Another method of isolation of halfordin was proposed by Sultana et al. [7]. The *n*-hexane extract of the aerial parts of *H. kendack* was subjected to vacuum liquid chromatography over silica gel. This approach allowed to obtain 416 mg of halfordin from 6.82 g of crude extract (yield 6%) [7]. The third reported approach of isolation of halfordin was proposed by Quek et al. [26]. After 72 h maceration of *Melilope latifolia* (DC.) T.G. Hartley (Rutaceae) bark with *n*-hexane, chloroform, and methanol, the extract was fractionated by column chromatography using step-gradient elution with *n*-hexane, mixtures of *n*-hexane and ethyl acetate, mixtures of ethyl acetate and methanol, and methanol. The process was time-consuming, the yield of that isolation was low, and it allowed to obtain 6.4 mg of halfordin from 17 g of extract [26]. The LLC method proposed in the current allowed the isolation of high-purity halfordin (11.2 mg, purity > 93%) from 270 mg extract in a short time (<20 min) and a single chromatographic step. Additionally, no pre-purification of the extract was needed before isolation. The process might be easily scale-up to an industrial scale by directly transferring the operating conditions to bigger column sizes.

Due to their small molecular weight and high lipophilicity, coumarins can easily penetrate the blood-brain barrier; therefore, they are good candidates for drugs with (CNS) activity. So far, the antiseizure effects of various coumarins have been reported in a series of in vivo assays with rodents or zebrafish [9,27,28,29,30]. Previously two other furanocoumarin derivatives, lucidafuranocoumarin A and bergamottin, were tested in the zebrafish PTZ-induced seizure model. Both compounds are linear furanocoumarins with a long chain in C5 of the coumarin ring; however, lucidafuranocoumarin A possesses an epoxy bridge in the aliphatic chain; this structural element was indicated as possibly responsible for the difference in the activity of bergamottin and lucidafuranocoumarin A [31]. Further systematic analysis of the behavioral antiseizure activity of a series of coumarin derivatives in the larval zebrafish PTZ model measurements allowed us to select compounds with very promising antiepileptic activity, what was further confirmed with pronounced antiepileptiform activity measured by LFP recordings from the zebrafish opticum tectum (midbrain). Molecular docking to the active center of GABA-transaminase of the most active C5-substituted coumarins: oxypeucedanin, oxypeucedanin hydrate, and notopterol, confirmed that coumarin derivatives containing a bulky substituent at the C5 position of the furochromenone ring showed significant antiseizure activity in the locomotor assay and antiepileptiform activity in LFP measurements. In contrast, an analogous bulky moiety substituted at the C8 position diminishes this activity [31]. The inhibition of seizures induced by PTZ suggests that the antiepileptic mechanism of halfordin is related to GABA. The affinity to the GABA_A_ receptor is structurally dependent, and compounds with substitution in both C5 and C8 positions exert more potent antiseizure activity than those with moieties only in C5 or C8 [27]. Halfordin is substituted in three positions—C3, C4, and C5; compared to the compounds substituted in both C5 and C8, the antiseizure activity lower of halfordin was lower, yet still significant.

To confirm the antiseizure activity of halfordin, the LFP assay was performed. The technique is similar to those used for decades in humans, though in zebrafish a single extracellular electrode is used. Zebrafish larvae at 7-dpf possess brain structures that allow seizure activity development. Not only do the larvae exhibit seizure-like behaviour, but abnormal electrical changes in CNS are also present [19,32]. Therefore, the method provides insight into electrical changes in the brain (optic tectum), which is a valuable tool for providing behavioural results.

To elucidate the mechanisms underlying the antiseizure activity, 11 genes were chosen for gene expression studies. The expression of only four genes was significantly changed. FBJ osteosarcoma oncogene (*c-fos*) is a proto-oncogene expressed rapidly and transiently in neurons following neuronal excitation, such as seizure episodes. Therefore, the expression level of *c-fos* has been considered an excellent parameter and widely used as a marker for neuronal activity. The use and validation of *c-fos* as a sensitive marker for investigating the anticonvulsant properties of several pharmacological compounds has been previously reported [19,33]. Previous studies indicate that *c-fos* in involved in many physiological and pathological processes, including proliferation, differentiation, transformation, and cellular death. It could be induced by many stimuli both external and internal, including hormones, growth factors and drugs. Studies have shown that expression of c-fos is associated with a variety of diseases, including cancer, neurodegenerative diseases, and inflammation [34,35].

Our results confirmed that *c-fos* expression is highly induced after PTZ treatment; however, a decrease in *c-fos* expression after halfordin treatment was not observed. The results in gene expression do not support the thesis about inhibition of neuronal activity suggested by the results obtained in behavioral and LFP experiments. Differences in *c-fos* may be observed in particular brain structures, (e.g., cerebrum), whereas whole-body expression of genes was tested in our experimental setup, but with no changes in expression observed. Thus, more subtle differences in regional *c-fos* expression may not be detected from whole animal expression studies. Another possible explanation is that protein levels are not always equal to levels of mRNA; therefore, the protein levels might differ from mRNA levels.

Brain-derived neurotrophic factor *(bdnf*) is a member of the neurotrophin family and plays an essential role in the CNS of mammals and zebrafish. BDNF is important for neurogenesis and neuronal maturation and is involved in learning and memory functions. It is widely distributed in the 7 dpf larval brain, and most abundant in the forebrain [36,37,38]. *Bdnf* is expressed in neurons, astrocytes, microglia, and neural areas prone to seizure—the hippocampus and entorhinal cortex. Our results confirm that levels of *bdnf* mRNA are increased during seizures induced by PTZ. The exact role of *bdnf* in epilepsy is still unknown. Studies indicate that *bdnf* reduces the inhibitory effect of the GABAergic system and boosts excitatory synapses of the glutamatergic system. In addition, the levels of *bdnf* are correlated with the severity of primary epilepsies (e.g., temporal lobe). Thus, it has been postulated that *bdnf* might be an indicator of seizure severity. On the other hand, prolonged infusion of *bdnf* downregulates and desensitizes tropomyosin receptor kinase B (TrkB) signalling pathways, possibly altering chloride transporter expression, which reduces neuronal excitability [39].

*Galn* is a gene encoding galanin, a 29–30 amino acid long neuropeptide, widely expressed in the central and peripheral nervous systems in many studied species [40], including zebrafish [41,42]. In the brain, galanin may function as an inhibitory neuromodulator/neurotransmitter or as a hypophysiotropic messenger in the anterior pituitary. Neuroanatomical localization and physiological properties of galanin suggest that this peptide may be involved in regulating seizures. Galanin is highly expressed in the hippocampus, the primary gateway for the propagation of seizure activity [43], and exerts a presynaptic inhibitory effect on glutamatergic transmission, inhibiting epileptic seizures [44,45,46]. Several studies have shown that galanin is involved in seizure regulation and can modulate epileptic activity in the brain [45,47]. *Galn* is a highly inducible neuropeptide, upregulated by many factors affecting the nervous system. A significant increase in *galn* expression is observed after peripheral nerve injury, with inflammation, in the basal forebrain in Alzheimer’s disease, during neuronal development, and after stimulation with estrogen. *Galn* has been found to regulate seizures, especially those in temporal lobe epilepsy. Transgenic mice with underexpressed *galn* show higher sensitivity to seizures than wild-type mice; transgenic mice with overexpressed *galn* exhibit inhibited progression of induced seizures [48].

In our study, changes in *galn* expression after PTZ administration were not observed, which is consistent with previous reports. [49] Galanin is also expressed in many tissues outside the nervous system, so it might be concluded that changes in brain expression are masked by expression in other organs, as was postulated for *c-fos*. It should also be mentioned that it is not clear whether galanin expression is increased during the seizures, with some studies finding no changes in galanin levels in particular brain regions [50].

Proenkephalin a (*penka*) is a gene encoding endogenous opioid preproprotein *proenkephalin a.* Enkephalins derived from *penka* are endogenous ligands for the opioid receptors, including delta opioid receptors [51]. Enkephalins regulate many vital functions, including pain, analgesia, and responses to stress and aggression [52]. There may be a link between stress and seizures, as enkephalins are upregulated in the hippocampus after exposure to stressors [53]. The activation of delta opioid receptors by enkephalins reduces seizure threshold, resulting in proconvulsive properties. At the same time, the activation of these receptors is linked to neuroprotection by preventing oxidative damage [53,54]. The PTZ treatment did not significantly changed the expression of *penka*, however we noticed upregulation after administration of halfordin. Many previous studies confirmes that furanocoumarins possess strong antioxidant activity, which might be linked with neuroprotection, however due to the lack of data considering *penka* expression and epilepsy, further research is required [55].

## 4. Materials and Methods

### 4.1. Chemicals

Analytical grade *n*-hexane, and methanol, dichloromethane were purchased from POCh (Gliwice, Poland), whereas LC grade methanol, acetonitrile, and formic acid were provided by J.T. Baker (Deventer, the Netherlands). Dimethyl sulfoxide (DMSO), sodium valproate (VPN) and pentylenetetrazole (PTZ), calcium chloride (CaCl_2_), sodium bicarbonate (NaHCO_3_), potassium phosphate monobasic (KH_2_PO_4_) and glucose were acquired from Merck (Poznań, Poland). 4-(2-Hydroxyethyl)-1-piperazineethanesulfonic acid (HEPES) buffer, sodium chloride (NaCl), potassium chloride (KCl) were purchased from POCh (Gliwice, Poland), magnesium sulfate (MgSO_4_), and calcium nitrate (Ca(NO_3_)_2_) from Chempur (Piekary Śląskie, Poland) and low melting agarose from ThermoFisher (Waltham, MA, USA).

### 4.2. Plant Material and Extraction

Leaves of *Halfordia kendack* (Montrouz.) Guillaumin were collected in May 2014 in Creek Pernod (S 22.18266/E 166.83945), Yaté Region (New Caledonia) by Edouard Hnawia (E.H.) and Mohammed Nour (M.N.) from the University of New Caledonia. A voucher specimen (EH015.37) has been deposited at the Medical University of Lublin (Poland). Dried and grounded leaves (56.41 g) were extracted with methanol (600 mL) in an ultrasound bath. After solvent removal, 12.76 g extract were obtained (yield 22.79%).

### 4.3. Liquid-Liquid Chromatography Isolation

#### 4.3.1. Liquid-Liquid Chromatography (LLC) Unit

The experiments were performed on a centrifugal partition chromatography (CPC) unit (model CPC-250) from Gilson (Middleton, WI, USA), with a total column volume of 250 mL, a maximum rotation speed of 3000 rpm, a maximum pressure drop of 100 bar and typical flow rate up to 15 mL/min. The unit was connected to a preparative PLC2250 LC system from Gilson with a DAD detector.

#### 4.3.2. Selection of Biphasic Solvent System (Shake Flask Experiments)

To select the suitable solvent system, different ratios of *n*-hexane, ethyl acetate, methanol, and water (HEMWat) were prepared and tested in shake-flasks, as follows: ~1 mg of the methanol leaf extract was added to 4 mL of the pre-equilibrated solvent system; after shaking, sample dissolution, and phase separation, 1 mL of the upper phase and 1 mL of the lower phase were taken, evaporated to dryness, re-dissolved in 1 mL methanol and analyzed by HPLC-DAD. The biphasic solvent system was chosen based on the partition coefficient (K_D_), calculated as the ratio between the peak area of the target compound in the upper phase and the peak area of the same compound in the lower phase.

#### 4.3.3. Preparation of the Biphasic Solvent System

The selected solvent system was prepared fresh before the experiments by mixing the corresponding solvent volumes in a separatory funnel, followed by vigorous shaking, phase separation, splitting the upper and lower phase into two different containers, and degassing in an ultrasound bath for 20 min.

#### 4.3.4. LLC Separation

Firstly, the column was filled with the upper stationary phase of the selected biphasic solvent system at a flow rate of 12 mL/min. Then, the rotational speed was set to 1700 rpm, and the lower mobile phase was pumped into the column at a flow rate of 10 mL/min (descending mode) until no more stationary phase eluted from the column (column equilibrium has been reached). The volume of the extruded stationary phase was measured and used to determine the stationary phase fraction (*S_F_*), calculated as the ratio between the stationary phase and the column volume. The methanol leaf extract (270 mg dissolved in the lower phase) was then injected into the column in a volume of 10 mL via an injection loop. The eluent stream was continuously monitored with the UV detector at 254 nm. Fractions (1 min) were collected for 50 min and analysed individually by HPLC-DAD.

#### 4.3.5. HPLC-DAD Analysis

A Shimadzu 20A series HPLC system (Shimadzu, Tokyo, Japan) coupled with an automatic degasser (DGU-20A 3R), quaternary pump (LC-20AD), auto-sampler (SIL-20 HT) and diode array detector (SPD-M20A) was used for the HPLC-DAD analyses. The separations were performed on a Phenomenex Gemini NX-C18 110A (250 mm × 4.6 mm, 5 μm) column with the mobile phase composed of water (A) and methanol (B). The following gradient was applied: 0 min—50% B, 5 min—60% B, 20 min—80% B, 25 min—100% B, and 26–35 min—50% B. The flow rate was 1 mL/min, the column temperature was 25 °C, the injection volume was 10 μL, and the DAD spectra were recorded at 254 and 300 nm.

#### 4.3.6. Structure Elucidation

An Agilent 1200 HPLC system (Agilent Technologies, Santa Clara, CA, USA) coupled with a degasser (G1379B), binary pump (G1312C), column oven (G1316B), auto-sampler (G1329A), DAD detector (G1315B) and mass spectrometer (G6530) was used for the HPLC-DAD-ESI-QTOF-MS/MS analyses. The separations were performed on a Zorbax Stable Bond RP-18 (150 mm × 2.1 mm, 3.5 μm) column with the mobile phase composed of 0.1% formic acid in water (A) and 0.1% formic acid in acetonitrile (B). The following gradient was applied: 7 min—35% B, 20 min—40% B, 42 min—65% B, 43–50 min—95% B. The flow rate was 0.2 mL/min, the column temperature was 20 °C, the injection volume was 10 μL, and the DAD spectra were recorded at 210, 254, 320, and 366 nm. The following MS parameters were used: *m/z* 100–1000; positive ionization mode; gas temperature 350 °C; N_2_ flow rate 12 L/min, nebulizer pressure 40 psi, skimmer 65 V, capillary voltage 4000 V, fragmentor 120 V, fixed collision energies 10 and 40 V. The acquired MS data were compared with the literature [7,8].

A Bruker Avance Neo 600 NMR spectrometer (Bruker BioSpin, Rheinstetten, Germany) supplied with a QCI 5 mm Cryoprobe and a SampleJet automated sample changer was used for the NMR analyses. The following NMR analyses were performed: 1D NMR (^1^H-NMR; ^13^C-NMR in CDCl_3_) and 2D NMR (correlation spectroscopy, COSY; heteronuclear single-quantum correlation, HSQC; heteronuclear multiple-bond correlation, HMBC; rotating-frame Overhauser enhancement spectroscopy, ROESY). The internal standard for ^1^H and ^13^C NMR was represented by CDCl_3_ (δ_H_ 7.26; δ_C_ 77.16).

Halfordin: UV (methanol) λ_max_ 210, 244 and 307 nm; HREIMS *m/z* 277.0702 [M + H]^+^ (calcd. For C_14_H_13_O_6_^+^, ∆ = 1.68 ppm); HRMS/MS(+) *m/z* 262.0463, 247.0227, 233.0433, 219.0285, 216.0407, 201.0173, 188.0457, 176.0099, 173.0299, 159.0434, 145.0271, 131.0486; ^1^H NMR (CDCl_3_, 600 MHz) δ 3.92 (3H, s, 3OCH_3_), 4.04 (3H, s, 5OCH_3_), 4.23 (3H, s, 4OCH_3_), 6.93 (1H, dd, *J* = 2.3, 1.0 Hz, H-3′), 7.24 (1H, d, *J* = 1.0 Hz, H-8), 7.59 (1H, d, *J* = 2.3 Hz, H-2′); ^13^C NMR (CDCl_3_, 151 MHz) δ 60.9 (3OCH_3_), 61.7 (4OCH_3_), 62.7 (5OCH_3_), 96.1 (CH-8), 104.5 (CH-3′), 106.9 (C-10), 118.7 (C-6), 130.2 (C-3), 145.4 (CH-2′), 149.6 (C-9), 150.6 (C-5), 156.4 (C-7), 157.2 (C-4), 160.4 (C-2). The NMR data are in agreement with those published by N. Sultana et al. [7].

### 4.4. Zebrafish Experiments

#### 4.4.1. Maintaining Zebrafish

Zebrafish (*Danio rerio*) stocks of the AB strain were maintained at 28 ± 0.5 °C on a 14/10 h light/dark cycle under standard aquaculture conditions, and fertilized eggs were collected via natural spawning. Embryos were reared under 24 h light conditions in embryo medium: 1.5 mM, pH 7.1–7.3, 17.4 mM NaCl, 0.21 mM KCl, 0.12 mM MgSO_4_, and 0.18 mM Ca(NO_3_)_2_ at 28.5 °C. For incubation, 6 days post-fertilization (dpf) and for all measurements 7 dpf larvae were used. The zebrafish experiments described here were approved by the Local Ethics Committee in Lublin, Poland (license no: 36/2022).

#### 4.4.2. Toxicity Assay

To evaluate the toxicity of compounds and extract, the maximum tolerated concentration (MTC) was determined. The MTC is defined as the maximum concentration that did not cause death and where no more than 2 out of 10 larvae exhibited any signs of locomotor impairment, including lack of touch response after an 18 h incubation period [16]. Each larva was checked under the microscope for signs of acute locomotor impairment: weak response upon a light touch of the tail with a fine needle [56,57], loss of posture, body deformation, bulging of the eyes out of their sockets, slow or absent heartbeat, and death [16]. Zebrafish larvae were incubated with different concentrations of extract and tested compounds. Incubations were performed at 28.5 °C in complete darkness for 18 h. Halfordin was tested over the concentration range of 5–200 µM, whereas *H. kendack* methanol leaf extract over the concentration range of 25–200 μg/mL.

#### 4.4.3. Antiepileptic Evaluation

6-dpf larvae were preincubated in 100 µL of 1% DMSO, 5 mM VPN, or tested compounds in concentrations of 25–100 μg/mL for *H. kendack* methanol leaf extract or 5–20 μM for halfordin for 18 h in individual wells of a 96-well plate at 28.5 °C in the dark, 10 larvae per condition. To 100 µL of embryo medium or tested compound/extract solution 100 µL of a 40 mM PTZ solution was added to obtain a final concentration 20 mM of PTZ in each well [17]. Larvae were habituated for 5 min in a dark chamber of an automated tracking device (ZebraBox system; Viewpoint, Lyon, France). Then, the locomotor activity without proconvulsant treatment, followed by locomotor activity with proconvulsant treatment were measured for 30 min in the dark. The total locomotor activity was quantified using ZebraLab software (Viewpoint, Lyon, France) and was expressed in “actinteg” units, which is the sum of all pixel changes detected during the time period defined for the experiment [17]. All tracking experiments were performed at least in triplicate.

#### 4.4.4. LFP Recordings

Recordings were obtained from the optic tectum of 7-dpf larvae as described [16]. Briefly, after 18 h incubation with 20 μM halfordin, the larvae were placed in 20 mM solution of PTZ in 24 well plate for 15 min. Larvae were embedded in a layer of 2% low melting agarose prior to 20 min LFP recording. The electrode was filled with artificial cerebrospinal fluid (2 mM KCl, 124 mM NaCl, 2 mM CaCl_2_, 2 mM MgSO_4_, 1.25 26 mM NaHCO_3_, mM KH_2_PO_4_, 10 mM glucose) and placed into optic tectum. Recordings were made in current clamp mode, at a 2 KHz frequency, with digital gain of 10, and filtering between 0.5 Hz–1 KHz. Seizure incidence was analysed using Clampfit 11.1.0.23 software (Molecular Devices, San Jose, CA, USA). The recordings were performed on at least 8 larvae.

#### 4.4.5. RNA Isolation, Reverse Transcription and Quantitative PCR

The experiment was performed on 6-dpf zebrafish larvae. Two Petri dishes of 60 larvae each were prepared at the same conditions, incubated with 20 μM halfordin. After 18 h incubation, one of these was treated with PTZ to obtain final concentration 20 mM and left for another 30 min incubation. After the incubation larvae from both conditions were separated into 3 Eppendorf tubes in each 20 larvae. Samples were fast frozen in −80 °C and then RNA was isolated using the Total RNA mini kit provided by A&A Biotechnology (Gdańsk, Poland) according to manufacturer’s instructions [58].

500 ng of RNA was reverse transcribed to produce cDNA using a PrimeScript RT Master Mix (Perfect Real Time) for qRT-PCR (TaKaRa, Saint-Germain-en-Laye, France) according to manufacturer’s instructions.

Quantitative PCR was performed using SYBR Green in accordance with the manufacturer’s protocol (SYBR Select Master Mix, Applied Biosystems, Foster City, CA, USA) on QuantStudio 3 Real-Time PCR System instruments (Applied Biosystems, Foster City, CA, USA). For each sample, the Ct-value was deducted from the Ct-value of a control sample and the fold change of gene expression was calculated and normalized to the expression levels of a reference gene -*ef1α* (*Elongation factor 1-alpha*). Results were analysed using the ΔΔCt method. Data shown are mean ± SEM, n = 3 experiments. Primer sequences are available in Supplementary Materials (Table S1).

### 4.5. Statistical Analysis

Statistical analysis was performed on GraphPad Prism 9.0.0. version (San Diego, CA, USA). The data were analysed by one-way or two-way analysis of variance (ANOVA) with repeated measures, followed by Tukey’s or Bonferroni’s post-hoc test, respectively. The results are presented as means  ±  standard error of the mean (SEM). Differences were considered statistically significant if the *p*-value was less than 0.05.

## 5. Conclusions

In this study, the furanocoumarin, halfordin, was isolated by liquid-liquid chromatography from the endemic plant *Halfordia kendack* and investigated for its antiseizure activity in the larval zebrafish PTZ seizure model. The locomotor activity was followed by EEG recordings from zebrafish optic tectum and gene expression. Both the locomotor and EEG assays confirmed the antiseizure potential of halfordin, although the gene expression studies did not reveal the mechanism of action of halfordin in the PTZ model. However, it showed that the exposition to halfordin induced the gene expression of *galn and penka*, which is worthy of further studies.

## Figures and Tables

**Table 1:** Partition coefficient (K_D_) values of halfordin from the *H. kendack* methanol leaf extract in different volume ratios of *n*-hexane/ethyl acetate/methanol/water (HEMWat).

No.	HEMWat	K_D_-Halfordin
1	1/1/1/1	2.54
2	6/5/6/5	1.20
3	3/2/3/2	0.69
4	2/1/2/1	0.36
5	5/2/5/2	0.24
6	3/1/3/1	0.20
7	4/1/4/1	0.14

## Data Availability

Not applicable.

## Supplementary Materials

The following supporting information can be downloaded at: https://www.mdpi.com/article/10.3390/ijms24032598/s1.
