# Supplementary material for: Evaluation of the Antiseizure Activity of Endemic Plant Halfordia kendack Guillaumin and Its Main Constituent, Halfordin, on a Zebrafish Pentylenetetrazole (PTZ)-Induced Seizure Model
Source: Int J Mol Sci. 2023 Jan 30;24(3):2598. doi: 10.3390/ijms24032598 (PMC9916433; doi:10.3390/ijms24032598)
Supplement: Supplementary file 1 [file ijms-24-02598-s001.zip › ijms-2175849-supplementary.pdf]

Table S1. Primer sequences

Gene	Forward 5′-3′	Reverse 5′-3′	Accession no/source
c-fos	GTGCAGCACGGCTTCACCGA	TTGAGCTGCGCCGTTGGAGG	NM_205569.1
bdnf	GGCGAAGAGCGGACGAATATC	AAGGAGACCATTCAGCAGGACAG	NM_131595.2
htr1aa	CAGAGCAGAGCAGCACAAG	TGGTCTGAGAGTTCTGGTCTAATC	NM_001123321.1
htr1b	GTGTCGGTGCTCGTGATG	CAGCCAGATGTCGCAGATG	NM_001128709.1
htr2b	GCTGCTCATTCTTCTGGTCAT	GTTAGTGGCGTTCTGGAGTT	NM_001044743.1
gabarapa	AGGATCCACTTGAGGGCTGA	CTTCTTCGTGATGCTCCTGGT	NM_001013260.1 /
gabarapb	TGTAAACAACGTCATTCCCCCT	ATCTTCTTCGTGATGTTCCTGGT	NM_001386387.1
galn	AAGGATACTCCCAGTGCAAGG	CTTTCCTGCCAGTCCGTGTT	NM_001346239.1
th1	GACGGAAGATGATCGGAGACA	CCGCCATGTTCCGATTTCT	NM_131149.1 /
penka	CTTTGAGCGCCTGTCTCGTG	TTCTAATGTGCAGGCCAGTGTG	NM_200083.2
penkb	TGGTGGCAGGAGTCTAAACG	TTGGACATCGCGGACATCAT	NM_182883.1
ef1	CTGGAGGCCAGCTCAAACAT	ATCAAGAAGAGTAGTACCGCTAGCATTAC	

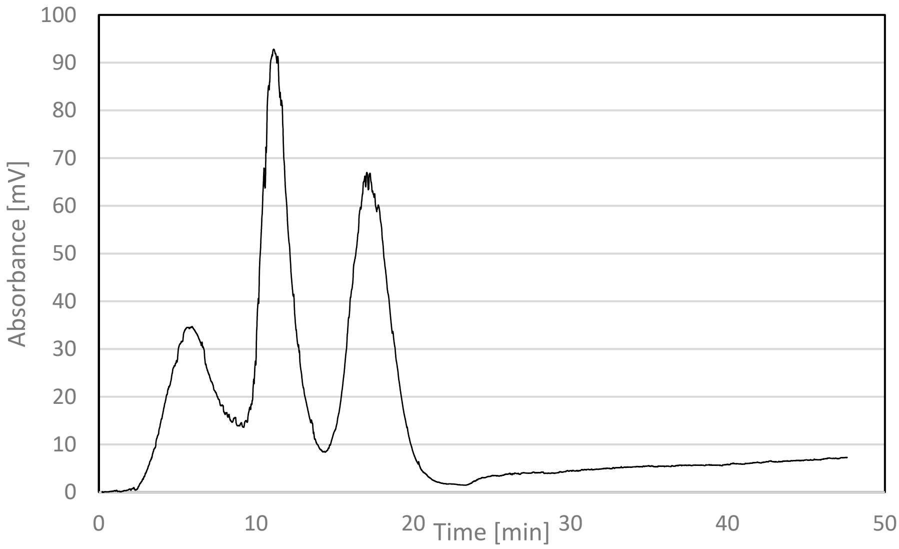

Figure S1. LLC chromatogram for the separation of halfordin from the methanol leaf extract of *H. kendack*. *Operating conditions*: Unit: CPC250; column volume 250 mL; solvent system *n*-hexane/ethyl acetate/methanol/water 3/1/3/1 (v/v/v/v); descending mode (lower phase as mobile phase); rotational speed 1700 rpm; flow rate 8 mL/min; sample 270 mg extract dissolved in 10 mL lower phase; injection volume 10 mL; UV 254 nm.
